# Biological Characteristics and Whole-Genome Analysis of Three Morphologically Distinct *Staphylococcus aureus* Phages

**DOI:** 10.3390/microorganisms14081635

**Published:** 2026-07-27

**Authors:** Yaqian Liang, Meihui Tian, Hengbai Zhang, Yize Guo, Jia Lu, Yang Zhao, Tianyi Zhao, Hui Zhang

**Affiliations:** College of Animal Science and Technology, Shihezi University, Shihezi 832000, China; 18409072772@163.com (Y.L.); 18699302505@163.com (M.T.);

**Keywords:** *Staphylococcus aureus*, bacteriophage, biological characteristics, whole-genome analysis, phylogeny

## Abstract

*Staphylococcus aureus* is an important zoonotic pathogen that threatens public health, food safety, and livestock production. Bacteriophages are promising antibacterial agents for controlling antimicrobial-resistant *S. aureus*, but their biological properties and genomic safety require systematic evaluation. In this study, three *S. aureus* phages, vB_SauL_202501, vB_SauM_202502, and vB_SauL_202503, were isolated from sewage samples and characterized by morphological observation, biological assays, whole-genome sequencing, and phylogenetic analysis. Transmission electron microscopy showed that vB_SauL_202501 and vB_SauL_202503 exhibited siphovirus-like morphology, whereas vB_SauM_202502 displayed myovirus-like morphology. Among the three phages, vB_SauM_202502 showed a relatively broader lytic spectrum and stronger sustained lytic activity in the spot assay. The optimal multiplicities of infection were 0.1, 0.1, and 0.01, respectively, and vB_SauL_202503 showed the shortest latent period, approximately 30 min. All three phages retained detectable infectivity at pH 5–10 and 4–37 °C, while vB_SauM_202502 maintained relatively high residual titers after heat treatment and ultraviolet irradiation. Genome analysis revealed double-stranded DNA genomes of 43,180–44,630 bp with GC contents of approximately 33.5%. No typical antimicrobial resistance genes or definitive auxiliary metabolic genes were identified. However, genes associated with lysogenic regulation, recombination, or immune modulation were detected in some phages, indicating that their lifestyles and application safety require further validation. These findings enrich *S. aureus* phage resources and provide a basis for the comparative characterization and safety assessment of candidate phages.

## 1. Introduction

*Staphylococcus aureus* (*S. aureus*) is an important Gram-positive zoonotic opportunistic pathogen that is widely distributed in natural environments as well as on the skin, in the nasal cavity, and on other mucosal surfaces of humans and animals. It can be transmitted through direct contact, environmental contamination, and the food chain, posing persistent threats to public health, food safety, and livestock and poultry production [1]. In humans, *S. aureus* can cause a wide range of infectious diseases, including skin and soft tissue infections, pneumonia, sepsis, and infective endocarditis, which may become life-threatening in severe cases. In addition, the heat-stable enterotoxins produced by *S. aureus* can induce acute gastroenteritis, making this bacterium one of the major causative agents of foodborne disease outbreaks [2,3]. In livestock and poultry production, *S. aureus* is associated with diseases such as bovine mastitis, avian arthritis, and porcine septicemia, resulting in reduced production performance, increased treatment costs, and potential risks to the quality and safety of animal-derived products. Moreover, it may further affect human health through animal-derived foods or contaminated farming environments [4]. For many years, antibiotics have been the primary means of controlling *S. aureus* infections. However, with the inappropriate use of antibiotics and the continuous increase in selection pressure, antimicrobial-resistant strains, such as methicillin-resistant *S. aureus* (MRSA) and vancomycin-resistant *S. aureus* (VRSA), have continued to emerge and disseminate. This has reduced the effectiveness of conventional antimicrobial control strategies and has also raised concerns regarding drug residues, environmental contamination, and disruption of the host microbiota [5]. Therefore, the development of safe and effective alternative antibacterial strategies with distinct mechanisms of action has become an important research direction for the prevention and control of *S. aureus* infections.

At present, newly reported alternative antibacterial approaches mainly include antimicrobial peptides [6], lysozymes [7], plant-derived antibacterial compounds [8], phage therapy [9], and nano-antibacterial materials [10]. Among these approaches, bacteriophages are viruses that infect bacteria, and under specific conditions, lyse host cells. As naturally occurring bacterial viruses, bacteriophages have gradually become important candidates in the development of alternative antibacterial strategies because of their relatively specific host recognition, strong antibacterial activity, and comparatively low risk of disturbing non-target microbiota. The host range of bacteriophages is mainly determined by the interaction between receptor-binding proteins and bacterial surface receptors, and phages usually exhibit selective infectivity toward specific bacterial species, strains, or clonal groups. Compared with broad-spectrum antibiotics, bacteriophages generally exert less impact on non-target microbial communities [11]. In addition, some phages can rapidly proliferate and lyse host bacteria under suitable host and environmental conditions, thereby exerting strong in vitro antibacterial activity [12]. Furthermore, bacteriophages differ from antibiotics in their antibacterial mechanisms. Although bacteria can develop phage resistance through mechanisms such as receptor modification, restriction-modification systems, and CRISPR-Cas systems, phages also possess a certain capacity for host adaptation and co-evolution. Therefore, strategies such as phage cocktails and combined phage-antibiotic applications may help reduce the risk of failure associated with a single antibacterial intervention and improve the control of antimicrobial-resistant bacterial infections [13]. Moreover, bacteriophages are not conventional chemical drugs and may have advantages in reducing drug residues and environmental burden. These features are consistent with the cross-sectoral control requirements for zoonotic pathogens and highlight their potential application value in medicine, food safety, and animal production [14].

To date, substantial progress has been made in studies of *S. aureus* phages, including phage isolation and identification, host range determination, environmental stability assessment, genome annotation, and comparative genomic analysis. These studies have provided an important foundation for the development and application of *S. aureus* phage resources [15]. Previous studies have shown that *S. aureus* phages from different sources and genetic backgrounds may differ in host range, infection kinetics, environmental adaptability, functional gene composition, and potential safety characteristics. Therefore, when evaluating newly isolated phage resources, it is necessary to integrate phenotypic assays with whole-genome information to comprehensively analyze their lytic activity, environmental stability, genomic composition, and safety-related features. In particular, for *S. aureus* phage isolates that originate from similar sources but differ in morphological parameters, biological properties, or genomic characteristics, parallel comparison can provide a more comprehensive understanding of their phenotypic and genetic features. Such comparisons can also provide a basis for subsequent candidate phage screening, combined application, and research on the control of antimicrobial-resistant *S. aureus*.

Based on this rationale, three *S. aureus* phage isolates were obtained from environmental samples in the present study. Their particle morphology was observed by transmission electron microscopy, and in accordance with current phage taxonomic terminology, they were described as siphovirus-like or myovirus-like morphotypes. Subsequently, the biological characteristics of the three phages were systematically evaluated, including host range, optimal multiplicity of infection, one-step growth curves, thermal stability, pH stability, and ultraviolet tolerance. In addition, whole-genome sequencing and bioinformatic analyses were performed to assess genome size, GC content, open reading frame composition, functional gene annotation, safety-related genes, and phylogenetic relationships. This study aimed to compare the biological properties and genomic characteristics of three *S. aureus* phage isolates, preliminarily evaluate their research value as candidate phage resources, and provide foundational data and resource reserves for phage-based control studies targeting antimicrobial-resistant *S. aureus*.

## 2. Materials and Methods

### 2.1. Collection and Pretreatment of Sewage Samples

The sewage samples used in this study were collected from Shihezi City and surrounding areas in Xinjiang Uygur Autonomous Region, China. A total of 35 sewage samples were collected and screened for the isolation of *S. aureus* phages. The sample types included wastewater from large-scale dairy farms, urban domestic sewage, and rural domestic sewage. Wastewater from large-scale dairy farms was collected from wastewater discharge outlets or temporary wastewater storage tanks. Urban domestic sewage was collected from the influent inlet of a municipal wastewater treatment plant in Shihezi City, whereas rural domestic sewage was collected from domestic sewage discharge sites in surrounding rural areas. Prior permission was obtained from the relevant administrative units before sampling. The sampling procedures were reviewed and approved by the Research Compliance Committee of the College of Animal Science and Technology, Shihezi University, and a research compliance statement was issued on 1 March 2025. All sampling sites were located outside protected areas, and no rare or endangered protected species were involved. All procedures complied with the Environmental Protection Law of the People’s Republic of China and the research management regulations of Shihezi University.

After collection, sewage samples were placed in sterile sampling bottles and transported to the laboratory under low-temperature conditions. The samples were first filtered through sterile gauze to remove large particulate impurities and then centrifuged at 6000 rpm for 30 min. The supernatant was collected, centrifuged again, and passed through 0.22 μm syringe filters for sterilization. The resulting filtrates were stored at 4 °C until further use. The 0.22 μm syringe filters were purchased from Wuhan Servicebio Technology Co., Ltd. (Wuhan, China).

### 2.2. Sources and Culture Conditions of Host Strains

The 101 *S. aureus* isolates used in this study were obtained from raw milk samples collected from large-scale dairy farms in the Shihezi region of Xinjiang, China. Species identification of the isolates was confirmed by whole-genome sequencing. The 101 *S. aureus* isolates used in the present study were the same strain collection previously characterized by our research group. Detailed information on their strain identifiers, sample sources, and multilocus sequence types has been reported in our previous publication [16] and is therefore not repeated here. The reference strains USA300 and ATCC 25923 were purchased from the American Type Culture Collection (ATCC, Manassas, VA, USA). A single purified colony of *S. aureus* was selected and inoculated into LB broth, followed by incubation at 37 °C with shaking at 180 rpm until the logarithmic growth phase was reached. The resulting bacterial cultures were used as host suspensions for phage enrichment, isolation, propagation, and subsequent biological characterization. LB medium was purchased from Qingdao Hope Bio-Technology Co., Ltd. (Qingdao, China).

### 2.3. Isolation, Purification, and Titer Determination of Phages

Phages were isolated using enrichment culture combined with the double-layer agar plate method. Briefly, sewage filtrate, logarithmic-phase host bacterial culture, and an appropriate volume of LB broth were mixed and incubated at 37 °C with shaking at 180 rpm. After incubation, the culture was centrifuged to remove bacterial cells and debris, and the supernatant was collected and filtered through 0.22 μm syringe filters to obtain the phage-enriched lysate. Subsequently, the phage-enriched lysate, logarithmic-phase host bacterial culture, and LB semi-solid agar medium cooled to approximately 55 °C were mixed and rapidly poured onto the surface of LB agar plates. After solidification, the plates were inverted and incubated at 37 °C for 8 h, and plaque formation was observed.

Single transparent plaques with clear morphology and regular margins were picked and resuspended in SM buffer. After centrifugation and filtration, the phages were further purified using the double-layer agar plate method. SM buffer was purchased from Suzhou Ulandy Biotechnology Co., Ltd. This purification procedure was repeated for five consecutive rounds until plaques with relatively uniform morphology and size were obtained. Finally, three phages were isolated and designated vB_SauL_202501, vB_SauM_202502, and vB_SauL_202503. The purified phages were propagated using the corresponding host strains to prepare phage lysates, and phage titers were determined by the double-layer agar plate method. According to the requirements of subsequent experiments, the phage lysates were diluted to the appropriate concentrations with SM buffer or LB broth and used as phage suspensions.

### 2.4. Morphological Analysis of Phages

The morphological characteristics of the three phages were observed using transmission electron microscopy (TEM). Each phage was mixed with its corresponding host bacterium at the optimal multiplicity of infection (MOI), inoculated onto double-layer agar plates, and incubated overnight at 37 °C. The plate surfaces were then rinsed with SM buffer to collect phage lysates. After filtration through 0.22 μm syringe filters, phage suspensions with a concentration of approximately 10^8^ PFU/mL were obtained. An aliquot of 10 μL of each phage suspension was applied to a carbon-coated copper grid and allowed to adsorb for 5 min. Excess liquid was carefully removed using filter paper, and the grid was negatively stained with 2% phosphotungstic acid (w/v, pH 7.0) for 60 s. After the excess stain was removed, the grid was air-dried at room temperature. Phage particles were observed and photographed using a Hitachi HT7800 transmission electron microscope (Hitachi, Tokyo, Japan) operated at an accelerating voltage of 80 kV. Measurements were calibrated according to the scale bar embedded in each micrograph using ImageJ v1.54u8 software. For each phage, at least three intact and clearly distinguishable particles from different microscopic fields were selected to measure the head diameter or width and tail length.

### 2.5. Host Range Determination

The host range of the three phages was determined using the spot assay. Briefly, 100 μL of logarithmic-phase *S. aureus* suspension at a concentration of 10^6^ CFU/mL was evenly spread onto the surface of LB agar plates. After the bacterial lawn was air-dried, 10 μL of purified phage suspension at a concentration of 10^8^ PFU/mL was spotted onto the surface of the lawn. An equal volume of SM buffer or LB broth was used as the negative control. Each sample was tested in triplicate. The plates were inverted and incubated at 37 °C, and lysis was observed at 4 h and 12 h. The clarity of the lysis zones and the presence or absence of regrown colonies around the lysis zones were recorded. A phage was considered to have lytic activity against a given strain under spot assay conditions if a clear and transparent lysis zone was formed.

### 2.6. Optimal Multiplicity of Infection Assay

The optimal multiplicity of infection was defined as the ratio of the initial number of phage particles, expressed as plaque-forming units (PFUs), to the number of host bacterial cells, expressed as colony-forming units (CFUs). Phage suspensions and logarithmic-phase host bacterial cultures were mixed at MOIs of 100, 10, 1, 0.1, and 0.01. For each MOI, 100 μL of the mixture was added to 10 mL of LB broth and incubated at 37 °C with shaking at 180 rpm for 4 h. After incubation, the mixture was centrifuged at 6000 rpm for 10 min. The supernatant was collected, filtered through 0.22 μm syringe filters, and used for phage titer determination. The experiment was performed in triplicate and used to determine the latent period and characterize the replication dynamics of each phage.

### 2.7. One-Step Growth Curve Assay

According to the optimal MOI of each phage, phage suspensions were inoculated into 20 mL of LB broth containing logarithmic-phase *S. aureus*. After mixing, the cultures were statically incubated at 37 °C for 10 min to allow phage adsorption to the host bacteria. The mixtures were then centrifuged at 6000 rpm for 10 min, the supernatants were discarded, and the bacterial pellets were resuspended in LB broth prewarmed to 37 °C. This washing step was repeated three times to remove unadsorbed free phages. The resuspended cultures were incubated at 37 °C with shaking at 180 rpm, and this time point was defined as 0 min. Samples of 500 μL were collected every 15 min for phage titer determination over a total incubation period of 180 min. The experiment was performed in triplicate and used to analyze the latent period, lysis period, and burst size of each phage.

### 2.8. Thermal Stability, pH Stability, and Ultraviolet Sensitivity Assays

Phage samples were aliquoted into 1 mL portions for subsequent stability assays. For the thermal stability assay, phage samples were incubated at 4 °C, 25 °C, 37 °C, 50 °C, and 60 °C, and phage titers were determined at 10, 20, 30, 40, 50, and 60 min. For the pH stability assay, the pH of LB broth was adjusted to values ranging from 1 to 14 using hydrochloric acid or sodium hydroxide. Then, 100 μL of phage suspension at a concentration of 10^8^ PFU/mL was added to each pH-adjusted medium. After incubation at 37 °C for 1 h, phage titers were determined. For the ultraviolet sensitivity assay, 10 mL of phage suspension was placed 20 cm below a 254 nm ultraviolet lamp. Samples were collected after 10, 20, 30, 40, 50, and 60 min of ultraviolet exposure for phage titer determination. All experiments were performed in triplicate.

### 2.9. Whole-Genome Sequencing, Annotation, and Phylogenetic Analysis of Phages

Whole-genome DNA libraries of the phages were constructed using the VAHTS^®^ Universal Plus DNA Library Prep Kit and sequenced on the Illumina NovaSeq 6000 platform using 150-bp paired-end sequencing (PE150). Raw sequencing reads were quality-filtered using SOAPnuke v2.0.5 to remove adapter-contaminated and low-quality reads. After quality control, 2,426,652, 2,296,893, and 2,441,597 clean read pairs were obtained for vB_SauL_202501, vB_SauM_202502, and vB_SauL_202503, respectively. De novo assembly was performed using MEGAHIT v1.1.2, and host-derived sequences were removed to obtain the target phage genome sequences. Clean reads were mapped back to the assembled genomes using BWA v0.7.17, and sequencing depth was calculated using SAMtools v1.8. The average sequencing depths of vB_SauL_202501, vB_SauM_202502, and vB_SauL_202503 were 6132.24×, 9877.88×, and 9727.54×, respectively. Each phage genome was recovered as a single target viral contig. Genome completeness and assembly quality were assessed using CheckV v1.0, together with assembly continuity and read-mapping depth. Genome circularity was evaluated using ccfind v1.4.5, with terminal overlaps of >50 bp and a sequence identity of >94% considered evidence of circularity.

Open reading frame (ORF) prediction and preliminary functional annotation of the three phage genomes were performed using Pharokka v1.7.2. In addition, functional assignments of phage-encoded proteins were further supported by PhageScope, BLASTx v2.15.0 searches against the PHROGs database, and annotation results from the UniProtKB, COG (COG2024), and KEGG (Release 118.2) databases. For the BLASTx searches against the PHROGs database, hits with an E-value of ≤1 × 10^−5^, amino acid sequence identity of ≥30%, and query coverage of ≥50% were retained. When multiple hits met these criteria, the hit with the lowest E-value and highest bit score was selected as the best-supported annotation. COG annotation was used only to describe the distribution of annotated genes among different functional categories. KEGG annotation was performed by assigning KEGG Orthology (KO) identifiers based on protein sequence alignment results and mapping them to corresponding pathways or functional modules. Antimicrobial resistance genes (ARGs) were screened using ResFinder v4.6.0 with minimum nucleotide sequence identity and gene coverage thresholds of 80% and 60%, respectively. Virulence-associated genes (VAGs) were predicted using ABRicate v1.0.1 based on the VFDB database with minimum nucleotide sequence identity and gene coverage thresholds of 80% and 80%, respectively.

Phylogenetic analyses were performed at both the whole-genome and conserved-gene levels. For whole-genome phylogenetic tree construction, the three phage genome sequences obtained in this study were used as query sequences for BLASTn v2.15.0 searches against the NCBI GenBank nr/nt database to identify homologous reference phage genomes. Reference sequences were selected according to the following criteria: the annotated host was Staphylococcus spp. or a staphylococcus-associated phage; the sequence represented a complete genome or a high-quality near-complete genome; the sequence showed high BLASTn similarity, high query coverage, and a low E-value with the phage genomes obtained in this study; and sequences with clear accession numbers, host sources, and taxonomic information were prioritized. Reference genomes repeatedly retrieved by different query sequences were retained only once. After screening and redundancy removal, a total of 52 homologous staphylococcal phage genome sequences were obtained. The three phage genomes from this study, the 52 reference staphylococcal phage genomes, and the outgroup sequence were submitted to the VICTOR online platform. Whole-genome distances were calculated using the Genome BLAST Distance Phylogeny (GBDP) method with the recommended parameters for prokaryotic viruses, and a balanced minimum evolution tree was constructed using the FASTME algorithm. Branch support was assessed using 100 pseudo-replicates, and the phylogenetic tree was visualized using the ggtree package.

For single-gene phylogenetic tree construction, the conserved phage terminase large subunit gene (terL) was used as the target gene. Pharokka was used to annotate the three phages in this study and homologous reference phage genomes downloaded from the NCBI GenBank database, and TerL protein sequences were extracted from the annotation results. Multiple sequence alignment was then performed using MAFFT, and low-quality alignment regions were removed using trimAl. A maximum likelihood (ML) phylogenetic tree was constructed from the trimmed sequences using IQ-TREE v3.0.1. The best-fit amino acid substitution model was automatically selected using the ModelFinder (MFP) strategy, and branch support was evaluated using 1000 ultrafast bootstrap replicates and the SH-aLRT test. The phylogenetic tree was visualized and refined using the iTOL online tool, and phage genome maps were generated using the Proksee online platform. The genome sequences of the three *S. aureus* phages vB_SauL_202501, vB_SauM_202502, and vB_SauL_202503 have been deposited in the GenBank database under accession numbers PZ371295, PZ379590, and PZ379591, respectively.

### 2.10. Data Analysis and Figure Preparation

Unless otherwise stated, all quantitative biological assays were performed as three independent biological replicates. For each biological replicate, host bacterial cultures and phage suspensions were independently prepared, and the complete experimental procedure was repeated under the same conditions. Data were summarized descriptively using WPS Office 2026 and are presented as the mean ± standard deviation. No inferential statistical comparisons or significance tests were performed. Experimental data graphs were generated using Origin 2024, and figure assembly and format optimization were performed using Adobe Illustrator 2025.

## 3. Results

### 3.1. Morphology and Host Range of Bacteriophages

**Figure 1:**
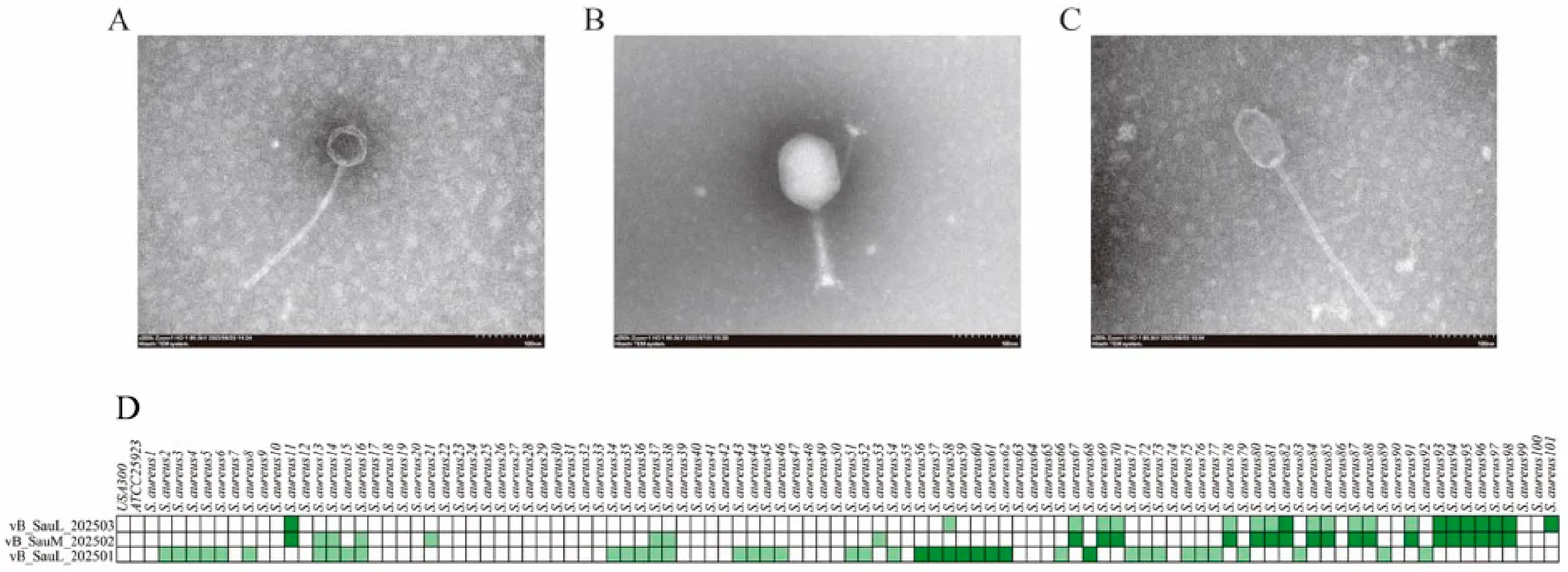
Transmission electron microscopy morphology and host range determination of three *S. aureus* bacteriophages. (**A**) Transmission electron microscopy image of vB_SauL_202501; (**B**) transmission electron microscopy image of vB_SauM_202502; (**C**) transmission electron microscopy image of vB_SauL_202503; (**D**) host range of the three phages against the tested *S. aureus* isolates determined using the spot assay. Light green indicates that a clear lysis zone was observed after 4 h, followed by bacterial regrowth after 12 h; dark green indicates that a clear lysis zone was maintained after 12 h without obvious regrowth; white indicates that no lysis was observed.

In this study, three bacteriophages were isolated from sewage samples using *S. aureus* isolates as host bacteria and were designated vB_SauL_202501, vB_SauM_202502, and vB_SauL_202503, respectively (Figure 1). After purification by the double-layer agar plate method, all three phages formed transparent plaques with relatively regular margins and diameters of less than 1 mm. Transmission electron microscopy showed that all three phages exhibited tailed phage particle morphology; however, differences were observed in head size, tail length, and tail structure. vB_SauL_202501 displayed a siphovirus-like morphology, with an average head diameter of 65.11 nm and an average tail length of 250.01 nm. vB_SauM_202502 exhibited a myovirus-like morphology, with an average head diameter of 115.09 nm and an average tail length of 116.10 nm, and a structure resembling a contractile tail sheath was observed. vB_SauL_202503 showed a siphovirus-like morphology, with an average head width of 24.61 nm and an average tail length of 304.22 nm. These morphological terms are used only to describe the particle characteristics observed under transmission electron microscopy and are not intended as formal family-level taxonomic assignments. Host range analysis showed that the three phages exhibited different degrees of lytic activity against the tested *S. aureus* isolates in the spot assay. Among them, vB_SauM_202502 showed a relatively broad lytic spectrum and stronger sustained lytic activity.

Figure 1A–C show the transmission electron microscopy images of phages vB_SauL_202501, vB_SauM_202502, and vB_SauL_202503, respectively. Figure 1D shows the host range of the three phages against the tested *S. aureus* isolates as determined by the spot assay. Light green indicates that a clear lysis zone was visible after 4 h of incubation, but regrown colonies appeared around the lysis zone after 12 h. Dark green indicates that a clear lysis zone was maintained after 12 h of incubation, with no obvious regrowth observed. White indicates that no lysis was observed under the experimental conditions used in this study.

### 3.2. Determination of the Optimal Multiplicity of Infection

**Figure 2:**
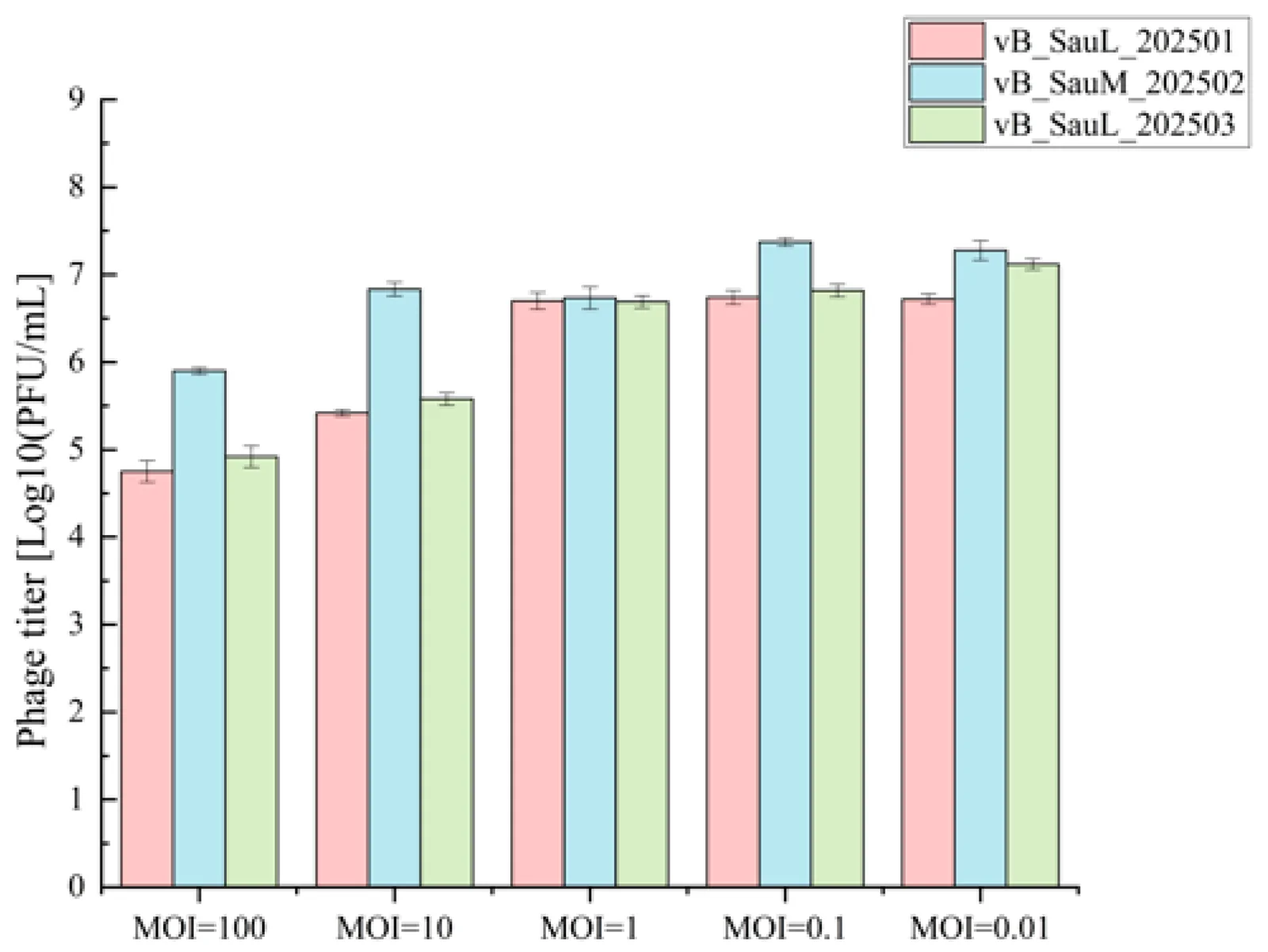
Changes in the titers of three *S. aureus* phages under different multiplicities of infection.

In this study, the phage titers of vB_SauL_202501, vB_SauM_202502, and vB_SauL_202503 were determined at multiplicities of infection (MOIs) of 100, 10, 1, 0.1, and 0.01 (Figure 2). The results showed that as the MOI decreased, the titers of all three phages initially increased and then fluctuated. Specifically, vB_SauL_202501 reached its highest titer at an MOI of 0.1, with a value of 6.741 ± 0.079 log_10_ PFU/mL. vB_SauM_202502 also reached its peak titer at an MOI of 0.1, showing the highest titer among the three phages, at 7.373 ± 0.045 log_10_ PFU/mL. In contrast, vB_SauL_202503 reached its maximum titer at an MOI of 0.01, with a value of 7.120 ± 0.061 log_10_ PFU/mL. Therefore, the optimal MOIs of vB_SauL_202501 and vB_SauM_202502 were both determined to be 0.1, whereas that of vB_SauL_202503 was determined to be 0.01.

The bar chart shows the phage titers of vB_SauL_202501, vB_SauM_202502, and vB_SauL_202503 at MOIs of 100, 10, 1, 0.1, and 0.01, expressed as log_10_ PFU/mL. Error bars represent standard deviations, and experiments were performed in triplicate.

### 3.3. One-Step Growth Characteristics of the Three Phages

**Figure 3:**
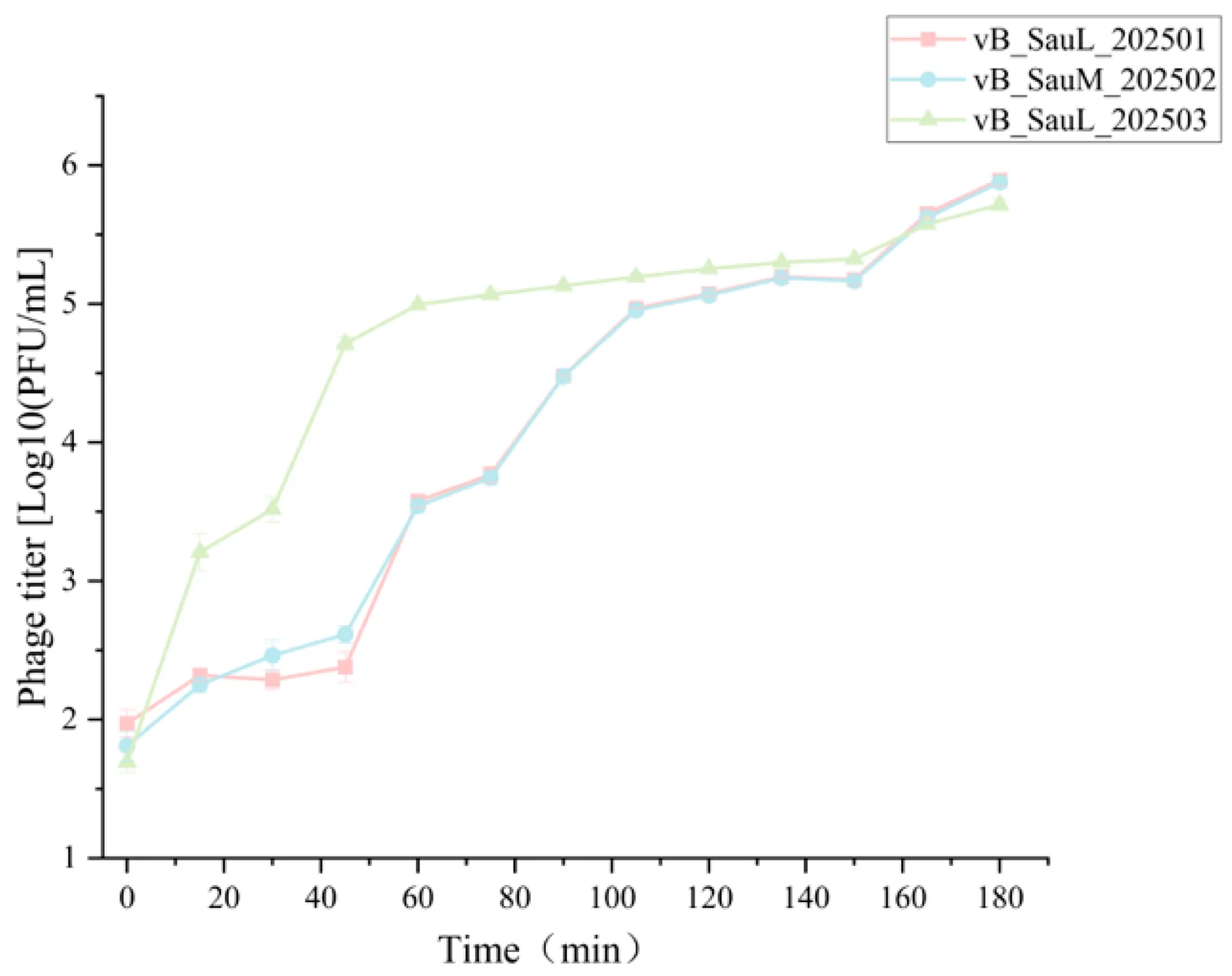
One-step growth curves of three *S. aureus* bacteriophages.

The one-step growth curves of vB_SauL_202501, vB_SauM_202502, and vB_SauL_202503 were determined in this study (Figure 3). The three phages exhibited distinguishable latent and rise phases, although their replication dynamics differed. The latent periods of vB_SauL_202501 and vB_SauM_202502 were both approximately 45 min. During 0–45 min, their titers changed only slightly, remaining within the ranges of 2.29–2.38 log_10_ PFU/mL and 1.81–2.62 log_10_ PFU/mL, respectively. After 45 min, the titers of both phages increased progressively and reached 5.89 ± 0.04 log_10_ PFU/mL and 5.88 ± 0.04 log_10_ PFU/mL at 180 min, respectively. In contrast, vB_SauL_202503 had a shorter latent period of approximately 30 min, after which its titer increased and reached 5.72 ± 0.04 log_10_ PFU/mL at 180 min. A further increase in the titers of all three phages was observed between 150 and 165 min. This late increase may have resulted from a secondary infection cycle or asynchronous release of progeny phages from infected cells and should not be interpreted as evidence of exceptionally rapid phage growth during the initial replication cycle.

The curves show changes in the titers of vB_SauL_202501, vB_SauM_202502, and vB_SauL_202503 from 0 to 180 min, expressed as log_10_ PFU/mL. Error bars represent standard deviations, and experiments were performed in triplicate.

### 3.4. pH Stability Assay of Phages

**Figure 4:**
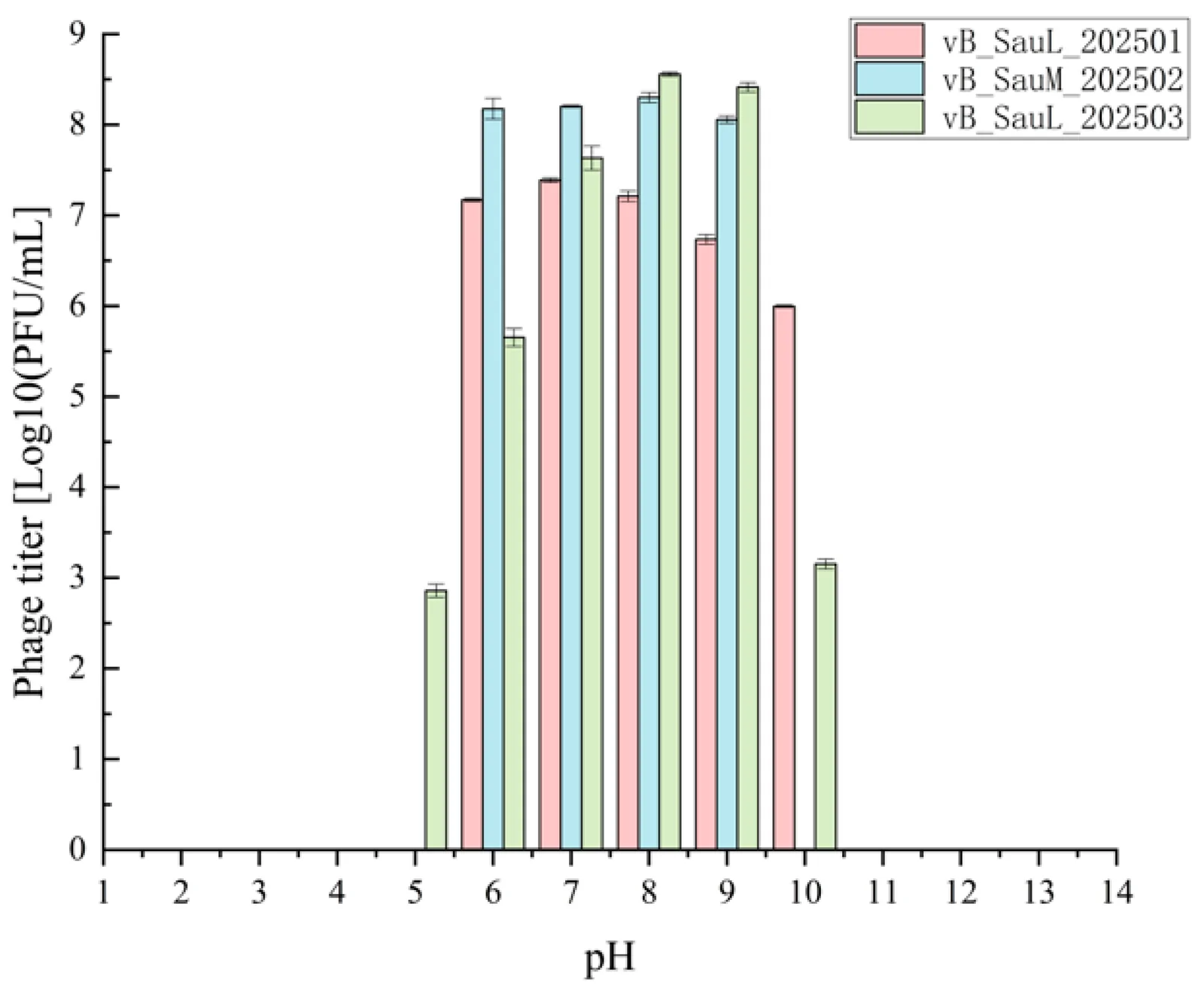
Changes in the titers of three *S. aureus* phages under different pH conditions.

The changes in the titers of vB_SauL_202501, vB_SauM_202502, and vB_SauL_202503 under pH conditions ranging from 1 to 14 were determined in this study (Figure 4). The results showed that all three phages were sensitive to extremely acidic and alkaline conditions. No infectivity was detected at pH < 5 or pH > 10, whereas different levels of activity were maintained within the pH range of 5–10. Specifically, vB_SauL_202501 retained detectable phage titers at pH 6–10 and reached its highest titer at pH 7, with a value of 7.39 ± 0.03 log_10_ PFU/mL. vB_SauM_202502 remained active at pH 6–9 and reached its maximum titer at pH 8, with a value of 8.30 ± 0.05 log_10_ PFU/mL. vB_SauL_202503 retained detectable infectivity across pH 5–10 and reached its peak titer at pH 8, with a value of 8.56 ± 0.02 log_10_ PFU/mL. Overall, the three phages showed better stability under neutral to weakly alkaline conditions, among which vB_SauL_202503 exhibited a relatively broader pH tolerance range.

The bar chart shows the changes in the titers of vB_SauL_202501, vB_SauM_202502, and vB_SauL_202503 under pH conditions ranging from 1 to 14, expressed as log_10_ PFU/mL. Under extreme pH conditions of pH < 5 or pH > 10, no infectivity was detected for any of the three phages. Error bars represent standard deviations, and experiments were performed in triplicate.

### 3.5. Ultraviolet Sensitivity Assay of Phages

**Figure 5:**
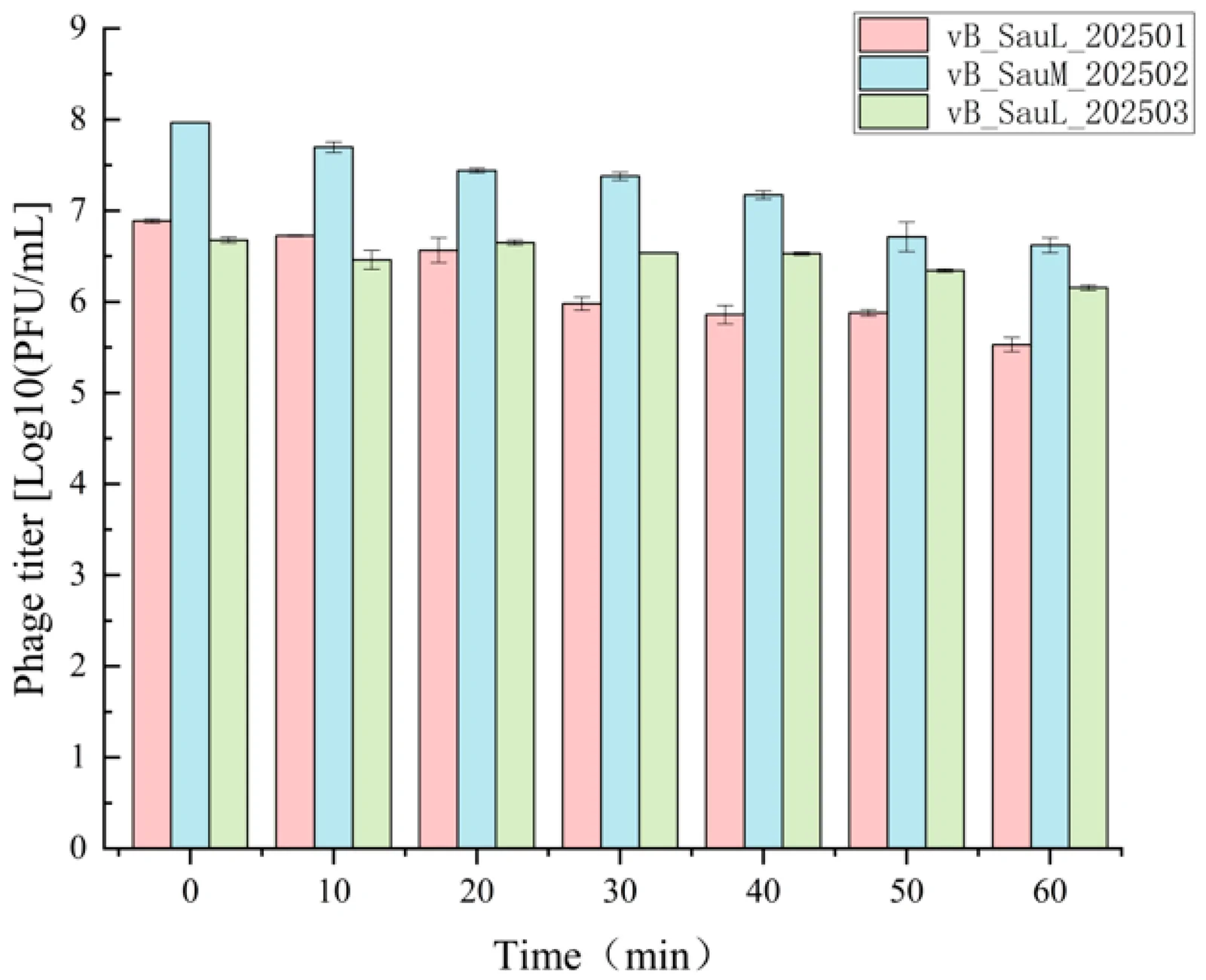
Changes in the titers of three *S. aureus* phages after ultraviolet irradiation.

The changes in the titers of vB_SauL_202501, vB_SauM_202502, and vB_SauL_202503 after exposure to 254 nm ultraviolet irradiation for 0–60 min were determined in this study (Figure 5). The results showed that the titers of all three phages decreased to varying degrees with prolonged ultraviolet exposure, indicating that ultraviolet irradiation reduced phage infectivity. Specifically, the initial titer of vB_SauL_202501 was 6.89 ± 0.02 log_10_ PFU/mL, which decreased to 5.53 ± 0.08 log_10_ PFU/mL after 60 min of irradiation. vB_SauM_202502 showed the highest initial titer, at 7.97 ± 0.00 log_10_ PFU/mL, and still maintained a titer of 6.62 ± 0.08 log_10_ PFU/mL after 60 min of irradiation, representing the highest residual titer among the three phages. The initial titer of vB_SauL_202503 was 6.68 ± 0.03 log_10_ PFU/mL and decreased to 6.15 ± 0.03 log_10_ PFU/mL after 60 min of irradiation, showing a relatively small reduction. Overall, vB_SauM_202502 maintained a relatively high absolute titer after ultraviolet exposure, whereas vB_SauL_202503 showed a smaller relative decrease in titer, suggesting that different phage isolates respond differently to ultraviolet irradiation.

The bar chart shows the changes in the titers of vB_SauL_202501, vB_SauM_202502, and vB_SauL_202503 after exposure to 254 nm ultraviolet irradiation for 0–60 min, expressed as log_10_ PFU/mL. Error bars represent standard deviations, and experiments were performed in triplicate.

### 3.6. Thermal Stability Assay of Phages

**Figure 6:**
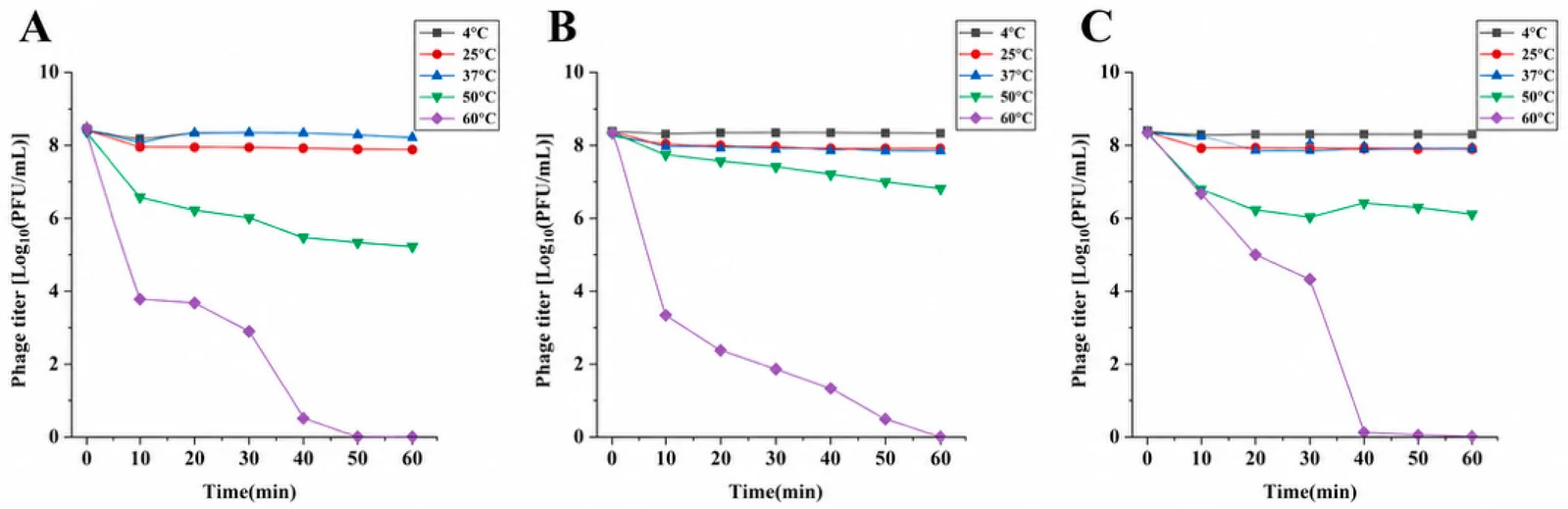
Changes in the titers of three *S. aureus* phages under different temperature conditions. (**A**) Changes in the titer of vB_SauL_202501 after treatment at 4 °C, 25 °C, 37 °C, 50 °C, and 60 °C for 0–60 min; (**B**) changes in the titer of vB_SauM_202502 after treatment at 4 °C, 25 °C, 37 °C, 50 °C, and 60 °C for 0–60 min; (**C**) changes in the titer of vB_SauL_202503 after treatment at 4 °C, 25 °C, 37 °C, 50 °C, and 60 °C for 0–60 min. Phage titers are expressed as log_10_ PFU/mL. Error bars represent standard deviations, and experiments were performed in triplicate.

The changes in the titers of vB_SauL_202501, vB_SauM_202502, and vB_SauL_202503 after treatment at 4 °C, 25 °C, 37 °C, 50 °C, and 60 °C for 0–60 min were determined to evaluate their thermal stability (Figure 6). The results showed that all three phages maintained high infectivity within the temperature range of 4–37 °C, with only slight fluctuations in titer after 60 min of treatment, suggesting good stability under refrigerated and conventional culture temperature conditions. Among them, vB_SauM_202502 still maintained a titer of 8.49 ± 0.06 log_10_ PFU/mL after treatment at 37 °C for 60 min.

At 50 °C and 60 °C, the titers of all three phages decreased with increasing treatment time, indicating that high-temperature treatment markedly reduced their infectivity. Specifically, the titer of vB_SauL_202501 decreased to 5.03 ± 0.03 log_10_ PFU/mL after treatment at 50 °C for 60 min, and no infectivity was detected after treatment at 60 °C for 40 min. vB_SauM_202502 still maintained a titer of 6.86 ± 0.05 log_10_ PFU/mL after treatment at 50 °C for 60 min, and no infectivity was detected after treatment at 60 °C for 50 min. The titer of vB_SauL_202503 was 5.93 ± 0.05 log_10_ PFU/mL after treatment at 50 °C for 60 min, and no infectivity was detected after treatment at 60 °C for 40 min. Overall, the three phages showed good stability at 4–37 °C, whereas their stability decreased under high-temperature conditions above 50 °C. Among them, vB_SauM_202502 retained a relatively higher residual titer after high-temperature treatment.

Figure 6A–C show the changes in the titers of vB_SauL_202501, vB_SauM_202502, and vB_SauL_202503, respectively, after treatment at 4 °C, 25 °C, 37 °C, 50 °C, and 60 °C for 0–60 min, expressed as log_10_ PFU/mL. Error bars represent standard deviations, and experiments were performed in triplicate.

### 3.7. General Genomic Features of the Phages

Whole-genome sequencing and assembly were performed for the three *S. aureus* phages, and their general genomic features are summarized in Table 1. The results showed that vB_SauL_202501, vB_SauM_202502, and vB_SauL_202503 were all assembled as double-stranded DNA (dsDNA) phage genomes. The genome sizes of the three phages were relatively similar. The genome length of vB_SauL_202501 was 43,180 bp, whereas those of vB_SauM_202502 and vB_SauL_202503 were 44,630 bp and 44,629 bp, respectively. The GC contents of the three phages were 33.47%, 33.58%, and 33.42%, respectively, showing the low-GC-content feature commonly observed in *S. aureus* phages. Gene prediction identified 61 open reading frames (ORFs) in both vB_SauL_202501 and vB_SauM_202502, and 62 ORFs in vB_SauL_202503. After preliminary annotation using Pharokka and supplementary functional assignment based on the UniProtKB database, 13, 15, and 15 proteins with relatively clear functional annotations were identified in the three phages, corresponding to functional annotation proportions of 21.31%, 24.59%, and 24.19%, respectively. The relatively low proportion of functionally annotated proteins is consistent with the common presence of numerous hypothetical or functionally unknown proteins in *S. aureus* phage genomes.

### 3.8. Functional Annotation and Analysis of Phage Genes

**Figure 7:**
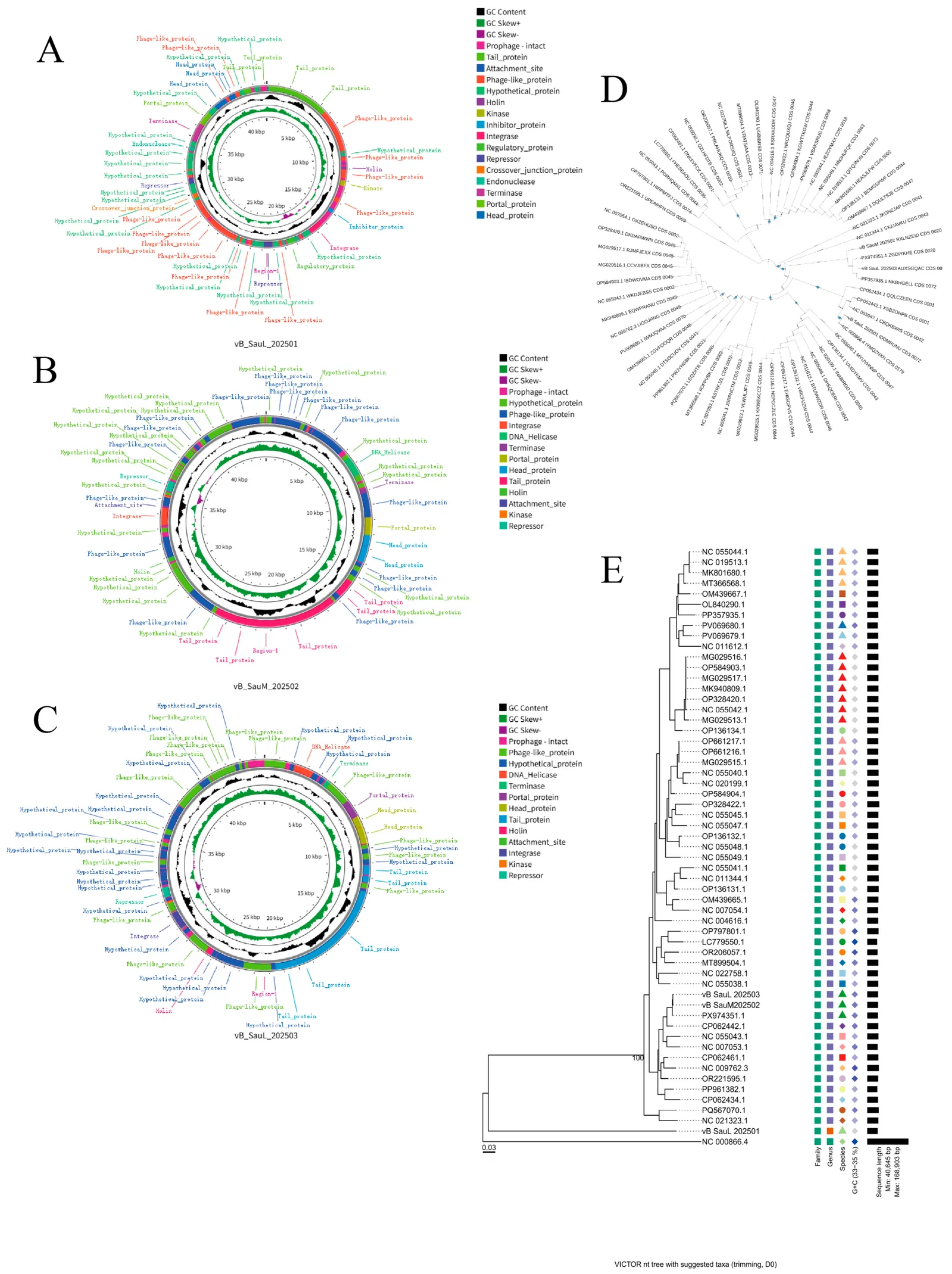
Genome maps and phylogenetic relationships of three *S. aureus* bacteriophages. (**A**–**C**) Circular genome maps of vB_SauL_202501, vB_SauM_202502, and vB_SauL_202503, respectively. Different colors represent the predicted gene functions shown in the legends, while the inner tracks represent GC content and GC skew. (**D**) Maximum-likelihood phylogenetic tree based on terminase large subunit (TerL) protein sequences. Branch support was evaluated using the SH-aLRT test and 1000 ultrafast bootstrap replicates. (**E**) Whole-genome phylogenetic tree constructed using the VICTOR/GBDP method. Numbers at the internal nodes indicate branch-support values obtained from 100 pseudo-replicates. The colors and symbols to the right of the tree represent the taxonomic and genomic characteristics indicated below the tree.

To analyze the functional gene composition of the three *S. aureus* phages (Figure 7A–C), the predicted proteins were functionally annotated using multiple databases, including COG, KEGG, UniProtKB, and PHROGs. The COG annotation results showed that some predicted proteins of the three phages could be assigned to functional categories associated with phage structure, DNA replication, recombination and repair, cell wall/cell membrane-related processes, and defense mechanisms. It should be noted that no statistical enrichment analysis was performed for the COG classification results in this study; therefore, these results are described only as the distribution of annotated genes across different functional categories and should not be interpreted as evidence of significant functional enrichment.

In vB_SauL_202501, several predicted proteins related to cell wall degradation, DNA metabolism, and regulation of the phage life cycle were annotated, including murein DD-endopeptidase/minor tail protein (ILJJJKFF_00001), integrase/site-specific recombinase XerD (ILJJJKFF_00013), phage repressor C (ILJJJKFF_00018), anti-repressor Ant (ILJJJKFF_00022), RecT recombinase (ILJJJKFF_00031), Holliday junction resolvase RusA (ILJJJKFF_00036), dUTPase (ILJJJKFF_00040), and terminase large subunit (ILJJJKFF_00050). In addition, several predicted proteins associated with host immune modulation or immune evasion were detected in this phage, including staphylokinase/Sak (ILJJJKFF_00009), chemotaxis inhibitory protein of *S. aureus*/CHIPS (ILJJJKFF_00011), and staphylococcal complement inhibitor/SCIN (ILJJJKFF_00012). These results suggest that although vB_SauL_202501 showed lytic activity in vitro, its genome harbors potential lysogenic regulatory elements and immune modulation-associated factors. Therefore, it should not be used as a natural therapeutic candidate phage without further safety evaluation.

The functional annotation results for vB_SauM_202502 and vB_SauL_202503 were mainly associated with phage genome packaging, particle structural assembly, host cell lysis, and DNA replication-related proteins. For example, vB_SauM_202502 encoded terminase large subunit (FKLEKIBE_00018), portal protein (FKLEKIBE_00019), major capsid protein (FKLEKIBE_00021), tape measure protein (FKLEKIBE_00030), endolysin LysH5 (FKLEKIBE_00040), integrase/site-specific recombinase XerD (FKLEKIBE_00041), and DNA polymerase (FKLEKIBE_00060). Similarly, vB_SauL_202503 encoded terminase large subunit (CIOPEDAG_00007), portal protein (CIOPEDAG_00008), major capsid protein (CIOPEDAG_00010), tape measure protein (CIOPEDAG_00019), endolysin LysH5 (CIOPEDAG_00029), integrase/site-specific recombinase XerD (CIOPEDAG_00030), phage repressor C (CIOPEDAG_00034), and DNA polymerase (CIOPEDAG_00049). These structural, packaging, lysis-related, and DNA metabolism-related modules represent common core functions of bacteriophages and may be present in both lytic and temperate phages. Therefore, they cannot be used alone as evidence for a strictly lytic lifestyle or genomic safety.

KEGG and UniProtKB annotations further showed that all three phages contained predicted proteins related to DNA replication, repair, recombination, and nucleotide metabolism, such as DNA primase/helicase, dUTPase, HNH endonuclease, RecB family exonuclease, and DNA polymerase. These results indicate that the three phages possess common core functional modules of bacteriophages, but they should not be interpreted as evidence that all three phages can stably complete an infection cycle or that they are strictly lytic phages. It should also be noted that although endolysin/LysH5-like proteins were annotated in all three phages and anti-holin-like proteins were detected in some genomes, typical holin genes were not clearly identified. Therefore, these phages cannot be described as possessing a complete holin-endolysin lysis system. The annotation results only suggest that their genomes contain predicted proteins associated with host cell wall degradation or lysis regulation, and the specific lysis mechanisms require further functional validation. Screening for antimicrobial resistance genes, virulence-associated genes, and auxiliary metabolic genes showed that no typical antimicrobial resistance genes or definitive auxiliary metabolic genes were detected in the three phages. However, immune modulation-associated predicted proteins, including Sak, CHIPS, and SCIN, were present in vB_SauL_202501, and lysogenic regulation- or recombination-related proteins, such as integrase, repressor, or recombinase, were detected in some phages. In addition, a p50-like/PVL ORF-50-like protein (FKLEKIBE_00059) was predicted in vB_SauM_202502.

### 3.9. Phylogenetic Analysis of Phages

To analyze the phylogenetic relationships between the three *S. aureus* phages and previously reported staphylococcal phages, phylogenetic trees were constructed based on whole-genome sequences and conserved terminase large subunit (TerL) protein sequences, respectively. The whole-genome phylogenetic tree constructed using the VICTOR platform showed that vB_SauL_202501, vB_SauM_202502, and vB_SauL_202503 each clustered with reported staphylococcal phage reference sequences from the NCBI GenBank database. Among them, vB_SauL_202501 and vB_SauL_202503 clustered in adjacent branches with several siphovirus-like staphylococcal phage reference sequences, whereas vB_SauM_202502 clustered in an adjacent branch with several myovirus-like staphylococcal phage reference sequences. Some key branches showed high bootstrap support values, indicating that these branches were well supported under the reference sequences, alignment data, and analytical model used in this study.

The maximum likelihood phylogenetic tree constructed based on TerL protein sequences showed a clustering pattern generally consistent with that of the whole-genome phylogenetic tree. Specifically, the TerL sequences of vB_SauL_202501 and vB_SauL_202503 clustered in adjacent branches with those of several siphovirus-like staphylococcal phage reference sequences, whereas the TerL sequence of vB_SauM_202502 clustered in an adjacent branch with those of several myovirus-like staphylococcal phage reference sequences. These results suggest that the three phages are phylogenetically related to previously reported staphylococcal phages and provide a reference for their relative phylogenetic positions.

Overall, whole-genome and TerL protein-based phylogenetic analyses showed that all three phages clustered to varying degrees with reported staphylococcal phage reference sequences and occupied distinct relative phylogenetic positions within the selected reference dataset. Because average nucleotide identity (ANI) analysis, whole-genome synteny analysis, and genome rearrangement analysis were not further performed in this study, no overinterpretation was made regarding the degree of sequence divergence, potential novel species status, or correspondence between morphological characteristics and molecular evolution among the three phages. These results are mainly intended to describe the relative phylogenetic relationships between the three phages and reference staphylococcal phages, and to provide a basis for future taxonomic and comparative genomic studies.

Figure 7A–C show the circular genome maps of vB_SauL_202501, vB_SauM_202502, and vB_SauL_202503, respectively. The outer rings display the predicted open reading frames (ORFs) and their functional annotation categories, with different colors representing different functional modules. The inner rings show the distributions of GC content and GC skew, respectively. Panel D shows the maximum likelihood phylogenetic tree constructed based on terminase large subunit (TerL) protein sequences, illustrating the relative phylogenetic relationships between the three phages and reference staphylococcal phage TerL sequences. Panel E shows the VICTOR/GBDP phylogenetic tree constructed based on whole-genome sequences, with the annotation information on the right indicating relevant genomic features and morphotype information of the reference phages. The phylogenetic tree results are intended only to describe the relative clustering relationships between the phages in this study and previously reported staphylococcal phage reference sequences, and should not be used as the sole basis for formal taxonomic nomenclature or novel species determination.

## 4. Discussion

In this study, three phage isolates with in vitro lytic activity were isolated from dairy farm wastewater, urban domestic sewage, and rural domestic sewage samples using *S. aureus* as the host bacterium. Their morphological characteristics, host range, infection kinetics, environmental stability, and genomic composition were systematically evaluated. The results showed that the three phages differed to some extent in particle morphology, lytic spectrum in the spot assay, one-step growth characteristics, environmental tolerance, and functional gene composition, suggesting that *S. aureus* phages from different sources or with different genomic backgrounds exhibit phenotypic and genetic diversity. Sewage environments harbor diverse bacterial communities and frequent microbial interactions, making them important sources for phage resource screening. Zhao et al. [17], in their study of the antimicrobial-resistant *S. aureus* phage SauPS-28, indicated that phage screening should not only focus on lytic activity but should also comprehensively evaluate host range, environmental stability, and genomic safety. Lerdsittikul et al. [15] isolated a Silviavirus phage from environmental samples and evaluated its anti-MRSA activity, further demonstrating that natural environments remain important reservoirs for the discovery of *S. aureus* phage resources. The present study further expands the collection of *S. aureus* phage resources from Xinjiang, China, and provides foundational materials for subsequent phage screening and comparative studies.

Host range and infection kinetics are important indicators for evaluating the application potential of bacteriophages. In this study, vB_SauM_202502 exhibited a relatively broad lytic spectrum in the spot assay, and clear lysis zones were maintained for some host strains after 12 h of incubation. In contrast, regrown colonies appeared around the lysis zones of some strains treated with vB_SauL_202501 or vB_SauL_202503, indicating differences in host range and sustained lytic performance among different phage isolates. It should be noted that the results of the spot assay cannot be fully equated with productive infection capacity and should be further validated by efficiency of plating (EOP) assays. The host range of phages is jointly influenced by receptor-binding proteins, host cell surface receptors, and host defense systems, and cannot be simply attributed to phage morphology. Li et al. [18] reported that phage vB_SA_STAP152 could lyse *S. aureus* strains covering multiple sequence types, and Wen et al. [19] reported that the broad-host-range phage SapYZU15 exhibited certain antibacterial effects in an infection model. These studies suggest that broad-host-range phages are of considerable interest for applied research. However, the breadth and stability of the host range still require comprehensive evaluation through standardized EOP assays, receptor recognition analysis, and in vitro and in vivo validation experiments.

The results of the infection kinetics showed that vB_SauL_202503 had a latent period of approximately 30 min under the experimental conditions used in this study, which was shorter than those of vB_SauL_202501 and vB_SauM_202502. The optimal MOIs of the three phages were 0.1, 0.1, and 0.01, respectively. These results indicate that different phage isolates may differ in adsorption, replication, and release processes. The MRSA phage reported by El-Tawab et al. [20] also exhibited a relatively low MOI and short latent period. However, MOI is an experimental parameter influenced by host bacterial density, culture conditions, and the experimental system, rather than an intrinsic property of the phage. Similarly, the latent period may vary depending on the host strain, medium, temperature, and other conditions. Therefore, the results of this study only indicate that the three phages exhibited different replication dynamics under the current in vitro experimental system, and a low MOI or short latent period should not be generalized as a universal feature of efficient proliferation in lytic phages.

Environmental stability determines the adaptability of phages during storage, transportation, and practical application. In this study, all three phages retained detectable infectivity at 4–37 °C and within the pH range of 5–10, whereas their titers decreased markedly after exposure to high temperatures above 50 °C, prolonged treatment at 60 °C, and extremely acidic or alkaline conditions. These results suggest that the three phages may be more suitable for use under refrigerated, room-temperature, or mild environmental conditions. Raveendran et al. [21] reported that a Silviavirus phage could tolerate a broader range of temperatures and pH conditions, and Li et al. [22] showed that vB_SA_STAP152 retained activity under relatively high temperatures and a wide pH range, indicating substantial differences in environmental tolerance among different phages. In the present study, vB_SauM_202502 retained a relatively high residual titer after treatment at 50 °C and ultraviolet irradiation, whereas vB_SauL_202503 showed a relatively smaller decrease in titer after ultraviolet exposure. These results indicate that different phages respond differently to heat treatment and ultraviolet irradiation. If these phages are to be applied in farming environments, food processing, or surface disinfection, their stability should be further evaluated in relation to matrix type, temperature, pH, ultraviolet exposure intensity, and treatment duration.

Whole-genome sequencing and functional annotation provide important information for evaluating phage resources. In this study, three double-stranded DNA phage genomes were assembled, with GC contents of approximately 33.5%. Their encoded proteins were mainly associated with common core phage functional modules, including structural assembly, genome packaging, DNA replication and repair, recombination, and host cell lysis. It should be emphasized that these core functional modules may be present in both lytic and temperate phages and therefore cannot be used alone as evidence for a strictly lytic lifestyle or genomic safety. Studies by Lu et al. [21] on phage StAP1 and by Zanaty et al. [23] on phage PSK both adopted an integrated strategy combining biological characterization with genomic analysis, indicating that functional annotation can provide useful references for phage screening. Nevertheless, comprehensive assessment should also include lysogen formation ability, transduction risk, and in vitro and in vivo safety evaluation.

No typical antimicrobial resistance genes or definitive auxiliary metabolic genes were detected in the three phages in this study. However, several predicted proteins related to recombination, integration, or lysogenic regulation were present in some phages. Specifically, vB_SauL_202501 encoded lysogenic regulation-related proteins such as phage repressor C and anti-repressor Ant, and also harbored predicted proteins related to immune modulation or immune evasion, including Sak, CHIPS, and SCIN. Phage repressor C was also detected in vB_SauL_202503. In addition, integrase/site-specific recombinase XerD and other recombination- or potential integration-related proteins were annotated in both vB_SauM_202502 and vB_SauL_202503. Studies by Naorem et al. [24], Wang et al. [25], and Liang et al. [15] have shown that the prophage composition of *S. aureus* is closely associated with strain origin, lineage background, and the distribution of virulence-associated genes. Monecke et al. [26] also reported that prophages in certain equine *S. aureus* lineages carried genes encoding an equine staphylococcal complement inhibitor and a putative kinase. Therefore, the formation of transparent plaques, in vitro lytic activity, and one-step growth curves cannot independently prove that a phage is strictly lytic. In particular, vB_SauL_202501 harbors lysogenic regulation-related proteins and immune modulation-associated factors, and thus should not be used as a natural therapeutic candidate phage without further safety evaluation or genetic modification. For future phage therapy or biocontrol applications, candidate phages lacking lysogenic regulatory modules, virulence-associated genes, and antimicrobial resistance genes should be prioritized, followed by lysogen formation assays, transduction risk assessment, and in vitro and in vivo safety validation.

Phylogenetic analysis showed that all three phages clustered with previously reported staphylococcal phage reference sequences and occupied distinct relative phylogenetic positions within the selected reference dataset. It should be noted that phylogenetic trees only reflect relative clustering relationships under specific datasets, alignment methods, and evolutionary models, and cannot independently prove formal taxonomic status, novel species identity, or the degree of sequence divergence. Modern phage taxonomy generally requires comprehensive evaluation based on whole-genome similarity, average nucleotide identity (ANI), VIRIDIC, or other genomic metrics. Because ANI analysis, whole-genome synteny analysis, and genome rearrangement analysis were not performed in this study, no overinterpretation was made regarding the degree of sequence divergence, genetic uniqueness, or correspondence between morphological features and phylogenetic positions among the three phages. Future studies may use progressiveMauve, EasyFig, VIRIDIC, or ANI analysis to further compare genomic conservation, sequence divergence, and genome structural variation among these phages.

Phage resistance is an important factor affecting the long-term efficacy of single-phage applications. Jurado et al. [14] indicated that *S. aureus* can develop phage resistance through mechanisms such as receptor alteration, restriction-modification systems, and CRISPR-Cas systems, thereby potentially reducing the effectiveness of single-phage interventions. Cui et al. [11] summarized phage cocktails and phage-antibiotic combinations as important strategies for limiting the emergence of phage resistance and also highlighted phage-derived endolysins as promising alternative antibacterial agents. The PHC-1 phage cocktail developed by Guo et al. [27] reduced bacterial load and alleviated inflammatory responses in a mouse mastitis model. Studies by Cho et al. [28] and Krömker et al. [29] also suggested that phages have potential applications in the prevention and control of bovine mastitis. In addition, Suchithra et al. [30] reported that the Kayvirus phage RuSa1 exhibited strong antibiofilm activity against *S. aureus*., and Soto Lopez et al. [31] proposed that phage endolysins may serve as biocontrol agents in the food industry. These studies provide useful directions for future EOP determination, phage combination screening, antibiofilm evaluation, and in vivo application validation based on the phages characterized in this study.

In summary, this study isolated three *S. aureus* phages with in vitro lytic activity from sewage samples and compared their morphological characteristics, biological properties, and genomic composition. The results showed that the three phages differed in host range, infection kinetics, environmental stability, and functional gene composition. However, their strictly lytic lifestyles, transduction risks, and application safety require further validation. Future studies should focus on EOP determination, lysogen formation assays, whole-genome similarity and synteny analyses, antibiofilm activity evaluation, and in vivo safety and efficacy validation to further clarify their potential application value in the control of antimicrobial-resistant *S. aureus*.

## 5. Conclusions

In this study, three bacteriophage isolates with in vitro lytic activity were isolated from sewage samples using *S. aureus* as the host bacterium. These isolates were designated as vB_SauL_202501, vB_SauM_202502, and vB_SauL_202503, respectively, and their morphological characteristics, biological properties, and genomic compositions were systematically evaluated. The results showed that the three phages differed to some extent in particle morphology, lytic spectrum in the spot assay, infection kinetics, environmental stability, and functional gene composition. Among them, vB_SauM_202502 exhibited a myovirus-like morphology, showed a relatively broad lytic spectrum in the spot assay, and maintained relatively high residual titers after heat treatment and ultraviolet irradiation. vB_SauL_202503 exhibited a siphovirus-like morphology, a shorter latent period, and a smaller decrease in titer after ultraviolet irradiation. These findings indicate that different phage isolates exhibit distinct in vitro biological characteristics.

Genomic analysis showed that no typical antimicrobial resistance genes or definitive auxiliary metabolic genes were detected in the three phages. Their encoded proteins were mainly associated with common core phage functional modules, including structural assembly, genome packaging, DNA replication and repair, recombination, and host cell lysis. However, predicted proteins related to lysogenic regulation, recombination, or immune modulation were detected in some phages. In particular, vB_SauL_202501 encoded lysogenic regulation-associated proteins, including phage repressor C and anti-repressor Ant, and also harbored immune modulation- or immune evasion-associated factors, such as staphylokinase (Sak), chemotaxis inhibitory protein of *S. aureus* (CHIPS), and staphylococcal complement inhibitor (SCIN). These findings suggest that vB_SauL_202501 should not be used as a natural therapeutic candidate phage without further safety evaluation or genetic modification. In addition, a p50-like/PVL ORF-50-like protein was predicted in vB_SauM_202502, but whether it possesses anti-CRISPR-related activity requires further experimental validation.

Overall, this study enriches the available *S. aureus* phage resources and provides foundational data for the phenotypic comparison, genomic characterization, and safety evaluation of different phage isolates. It should be noted that this study was mainly based on in vitro experiments and genome-based predictive analyses. Therefore, the results cannot directly demonstrate that all three phages are strictly lytic, nor can they directly support their use in therapeutic or practical biocontrol applications. Future studies should further include the efficiency of plating assays, lysogen formation assays, transduction risk assessment, whole-genome similarity and synteny analyses, in vivo safety and efficacy validation, and evaluation of multi-phage combination strategies to clarify their potential application value in the control of antimicrobial-resistant *S. aureus*.

## Figures and Tables

**Table 1:** General genomic features of three morphologically distinct *S. aureus* phages.

Item.	vB_SauL_202501	vB_SauM_202502	vB_SauL_202503
Total base (bp)	43,180	44,630	44,629
GC content (%)	33.47	33.58	33.42
Total gene number	61	61	62
Genome type	ds DNA	ds DNA	ds DNA
Taxonomy	Staphylococcus phage	Staphylococcus phage	Staphylococcus phage
UniProtKB	13	15	15
Gene annotation rate (%)	21.31	24.59	24.19

## Data Availability

The complete genome sequences of the three *S. aureus* phages vB_SauL_202501, vB_SauM_202502, and vB_SauL_202503 generated in this study have been deposited in the NCBI GenBank repository under the accession numbers PZ371295, PZ379590, and PZ379591, respectively. All genome sequence data are publicly available as of May 11, 2026. All relevant raw data and analytical datasets supporting the findings presented in this study are included in the article. Further inquiries can be directed to the corresponding authors.

## References

[1] Nunes Nascimento J.C., Alvarenga V.O., Paulino B.N., de Cerqueira e Silva A.B., Matos J.R., Rosário I.L.D.S., Andrade I.H.P., Silva J.G., Costa M. (2026). *Staphylococcus aureus* in food safety: Antimicrobial resistance, detection technologies, and future perspectives. Crit. Rev. Food Sci. Nutr..

[2] Cheung G.Y.C., Bae J.S., Otto M. (2021). Pathogenicity and virulence of *Staphylococcus aureus*. Virulence.

[3] Campos B., Pickering A.C., Rocha L.S., Aguilar A.P., Fabres-Klein M.H., de Oliveira Mendes T.A., Fitzgerald J.R., de Oliveira Barros Ribon A. (2022). Diversity and pathogenesis of *Staphylococcus aureus* from bovine mastitis: Current understanding and future perspectives. BMC Vet. Res..

[4] Xiang Y., Li Z., Liu C., Wei Z., Mo X., Zhong Y., He R., Liang Z., He Y., He J. (2025). Pulsatilla chinensis extract alleviate *Staphylococcus aureus* induced mastitis in mice by regulating the inflammatory response and gut microbiota. Front. Vet. Sci..

[5] Mlynarczyk-Bonikowska B., Kowalewski C., Krolak-Ulinska A., Marusza W. (2022). Molecular mechanisms of drug resistance in *Staphylococcus aureus*. Int. J. Mol. Sci..

[6] Chen X., Yang J., Qu C., Zhang Q., Sun S., Liu L. (2024). Anti-*Staphylococcus aureus* effects of natural antimicrobial peptides and the underlying mechanisms. Future Microbiol..

[7] Belete M.A., Tadesse S., Tilahun M., Alemayehu E., Saravanan M. (2024). Phage endolysins as new therapeutic options for multidrug resistant *Staphylococcus aureus*: An emerging antibiotic-free way to combat drug resistant infections. Front. Cell. Infect. Microbiol..

[8] Kashi M., Noei M., Chegini Z., Shariati A. (2024). Natural compounds in the fight against *Staphylococcus aureus* biofilms: A review of antibiofilm strategies. Front. Pharmacol..

[9] Liu K., Wang C., Zhou X., Guo X., Yang Y., Liu W., Zhao R., Song H. (2024). Bacteriophage therapy for drug-resistant *Staphylococcus aureus* infections. Front. Cell. Infect. Microbiol..

[10] Joshi D.C., Chavan M.B., Tiwari S.W., Devi S. (2025). Advances in combating antimicrobial resistance in MRSA: A comprehensive review on nanotechnology and phage therapy. Antonie Van. Leeuwenhoek.

[11] Cui L., Watanabe S., Miyanaga K., Kiga K., Sasahara T., Aiba Y., Tan X.E., Veeranarayanan S., Thitiananpakorn K., Nguyen H.M. (2024). A comprehensive review on phage therapy and phage-based drug development. Antibiotics.

[12] Fortaleza J.A.G., Ong C.J.N., De Jesus R. (2024). Efficacy and clinical potential of phage therapy in treating methicillin-resistant *Staphylococcus aureus* (MRSA) infections: A review. Eur. J. Microbiol. Immunol..

[13] Tarasenko A., Papudeshi B.N., Grigson S.R., Mallawaarachchi V., Hutton A.L.K., Warner M.S., Barr J.J., Iredell J., Eijkelkamp B., Edwards R.A. (2025). Reprogramming resistance: Phage-antibiotic synergy targets efflux systems in ESKAPEE pathogens. mBio.

[14] Jurado A., Fernández L., Rodríguez A., García P. (2022). Understanding the mechanisms that drive phage resistance in staphylococci to prevent phage therapy failure. Viruses.

[15] Lerdsittikul V., Apiratwarrasakul S., Atithep T., Withatanung P., Indrawattana N., Pumirat P., Chaiwattanarungruengpaisan S., Thongdee M. (2024). Isolation and characterisation of a novel *Silviavirus* bacteriophage promising antimicrobial agent against methicillin-resistant *Staphylococcus aureus* infections. Sci. Rep..

[16] Liang Y., Wang W., Guo Y., Tian M., Wang J., Hao H. (2025). Study on genomic diversity, prophage distribution of bovine-derived *Staphylococcus aureus* and their association with antimicrobial resistance. Microorganisms.

[17] Zhao P., Zhao W., Zhai X., He Y., Shu W., Qiao G. (2024). Biological characterization and genomic analysis of a novel methicillin-resistant *Staphylococcus aureus* phage, SauPS-28. Microbiol. Spectr..

[18] Li Y., Dai J., Wu S., Rong D., Huang J., Zhao M., Zhang J., Ye Q., Gu Q., Zhang Y. (2025). The food application of a novel *Staphylococcus aureus* bacteriophage vB_SA_STAP152 and its endolysin LysP152 with high enzymatic activity under cold temperature. Food Microbiol..

[19] Wen H., Zhou W., Wu Y., Li Y., Zhu G., Zhang Z., Gu X., Wang C., Yang Z. (2023). Effective treatment of a broad-host-range lytic phage SapYZU15 in eliminating *Staphylococcus aureus* from subcutaneous infection. Microbiol. Res..

[20] El-Tawab A.K.A., Othman B.A., Sharaf A., El-Masry S.S., El-Arabi T.F. (2024). Characterization and complete genome sequence of highly lytic phage active against methicillin-resistant *Staphylococcus aureus* (MRSA) isolated from Egypt. Virol. J..

[21] Raveendran K., Sharaf S., D. A.L., K. R., Joseph T.C., Thangaraj R.S., Sivam V., Parida S., Hegde N.R., Shashidhar R. (2025). Novel *Silviavirus* phages with broad-host-range activity against methicillin resistant *Staphylococcus aureus* from seafood: Comprehending the phenotypic and genotypic variability. Folia Microbiol..

[22] Lu Y., Lu Y., Li B., Liu J., Wang L., Zhang L., Li Y., Zhong Q. (2023). StAP1 phage: An effective tool for treating methicillin-resistant *Staphylococcus aureus* infections. Front. Microbiol..

[23] Zanaty A.A., Dishisha T., El-Sayed-Ahmed M.A.E., Abdel-Fattah M.M., Ahmed K.A., Abdelkader K. (2025). Characterization, genomic analysis and preclinical evaluation of the lytic Staphylococcus bacteriophage PSK against methicillin-resistant *Staphylococcus aureus* wound isolate. Ann. Clin. Microbiol. Antimicrob..

[24] Naorem R.S., Goswami G., Gyorgy S., Fekete C. (2021). Comparative analysis of prophages carried by human and animal-associated *Staphylococcus aureus* strains spreading across the European regions. Sci. Rep..

[25] Wang Z., Peng X., Hülpüsch C., Khan Mirzaei M., Reiger M., Traidl-Hoffmann C., Deng L., Schloter M. (2024). Distinct prophage gene profiles of *Staphylococcus aureus* strains from atopic dermatitis patients and healthy individuals. Microbiol. Spectr..

[26] Monecke S., Burgold-Voigt S., Feßler A.T., Krapf M., Loncaric I., Liebler-Tenorio E.M., Braun S.D., Diezel C., Müller E., Reinicke M. (2025). Characterisation of *Staphylococcus aureus* strains and their prophages that carry horse-specific leukocidin genes lukP/Q. Toxins.

[27] Guo M., Zhang Y., Wu L., Xiong Y., Xia L., Cheng Y., Ma J., Wang H., Sun J., Wang Z. (2024). Development and mouse model evaluation of a new phage cocktail intended as an alternative to antibiotics for treatment of *Staphylococcus aureus*-induced bovine mastitis. J. Dairy Sci..

[28] Cho J.H., Lee G.M., Ko S., Kim Y., Kim D. (2025). Characterization and therapeutic potential of newly isolated bacteriophages against Staphylococcus species in bovine mastitis. J. Virol..

[29] Krömker V., Leimbach S., Tellen A., Wente N., Schmidt J., Lehnherr H., Nankemann F. (2026). Randomized, negative-controlled pilot study on the treatment of intramammary *Staphylococcus aureus* infections in dairy cows with a bacteriophage cocktail. Antibiotics.

[30] Suchithra K.V., Hameed A., Rekha P.D., Stothard P., Arun A.B. (2025). A novel Kayvirus species phage RuSa1 removes biofilm and lyses multiple clinical strains of methicillin resistant *Staphylococcus aureus*. Sci. Rep..

[31] Soto Lopez M.E., Mendoza-Corvis F., Salgado-Behaine J.J., Hernandez-Arteaga A.M., González-Peña V., Burgos-Rivero A.M., Cortessi D., Vidigal P.M.P., Pérez-Sierra O. (2025). Phage endolysins as an alternative biocontrol strategy for pathogenic and spoilage microorganisms in the food industry. Viruses.

